# Convulsions in a Bear

**Published:** 1882-01

**Authors:** 


### X.—CONVULSIONS IN A BEAR.

The Superintendent of the Zoölogical Garden, Elberfeld, Germany, describes, in *Der Zoölogische Garten*, the sickness of a brown bear, as follows:

The first symptoms were a loss of appetite, taking no nourishment, with the exception of a piece of sugar, the eating of which seemed to cause it pain. A severe diarrhœa followed, while the eyes became expressionless and the nose hot and dry. On the second day it was suddenly attacked with violent cramps, which seemed to affect the whole body. These spasms occurred every hour. The symptoms seemed to indicate poisoning by strychnia were it not for the severe diarrhœa. It was noticed that it tried to bite the iron bars of its cage, and this lead to the idea that the nerves of the teeth were affected. The remedy prescribed was 2 gramms of bromide of potash, mixed with pulverized sugar, five times every two hours. This gave relief.

[This is certainly an interesting case, but we do not see why the diarrhœa contra indicates strychniæ poisoning, for it is a well-known fact that this is one of our strong holds in stimulating peristaltic action. The convulsions may have been due to indigestion. Possibly the animal would have recovered as quickly without the bromide.—ED.]

